# Horizontal rectus muscle insertion sites in patients with infantile esotropia and acute acquired concomitant esotropia

**DOI:** 10.1016/j.heliyon.2025.e41882

**Published:** 2025-01-16

**Authors:** Shuyuan Liao, Yun Wen, Qingqing Ye, Linxing Chen, Tao Shen

**Affiliations:** a State Key Laboratory of Ophthalmology, Zhongshan Ophthalmic Center, Sun Yat-sen University, Guangdong Provincial Key Laboratory of Ophthalmology and Visual Science, Guangdong Provincial Clinical Research Center for Ocular Diseases, Guangzhou, 510060, China; b Zhongshan Medical School, Sun Yat-sen University, Guangzhou, 510000, China

**Keywords:** Medial rectus muscle, Lateral rectus muscle, Infantile esotropia, Acute acquired concomitant esotropia, Limbus-insertion distance, Insertion width

## Abstract

**Purpose:**

To investigate the characteristics of horizontal rectus muscle insertion sites in patients with infantile esotropia (IE) and acute acquired concomitant esotropia (AACE).

**Methods:**

The medical records of 166 IE patients and 261 AACE patients who underwent horizontal rectus muscle surgeries were reviewed retrospectively, including age at surgery, spherical equivalent (SE), and angle of esodeviation at near and distance. The limbus-insertion distances (LID) and widths of the medial rectus (MR) and lateral rectus (LR) muscles were measured intraoperatively using a caliper. The insertion parameters and clinical features were compared between different ages and types of esotropia, and a correlation analysis was performed between these variables.

**Results:**

In IE patients under 12 years old, the MR LID was positively correlated with age, whereas the LR LID was negatively correlated with the angle of esodeviation at distance. In IE patients over 12 years old, the LR width was negatively correlated with the angle of esodeviation at near. In AACE patients, the MR LID was negatively correlated with the angle of esodeviation at distance, whereas the LR width was negatively correlated with the SE.

**Conclusions:**

The LID and width of the horizontal rectus muscle insertions were not significantly different in age-matched patients with IE and AACE. In IE patients, the LR insertion parameters were correlated with the angle of esodeviation, but in AACE patients, the MR LID was correlated with the angle of esodeviation. The clinical significance of these findings still needs to be investigated further.

## 1 Introduction

Infantile esotropia (IE), first reported in 1961 [1], is characterized by an early onset of esotropia before six months of age and a large constant angle of esodeviation in the absence of neurologic disorders [2,3]. On the other hand, acute acquired concomitant esotropia (AACE) was first defined by Burian and Miller in 1958 [4], and characterized by the acute and late onset of esotropia with acquired diplopia, which usually occurs in older children and adults [5]. The estimated incidence of IE is approximately 0.1–10.% [6], while the incidence of AACE has continued to increase in recent years [7,8]. The etiology of both IE and AACE is still unclear, and the correlation between horizontal rectus muscle insertion parameters and the horizontal strabismus is still controversial. A previous study reported the medial rectus (MR) width was associated with the angle of esodeviation in patients with concomitant esotropia [9]. However, other research showed no significant difference between different types of strabismus and no correlation between the MR limbus-insertion distance (LID) and the angle of esodeviation in patients with IE [10,11]. Therefore, the characteristic of lateral rectus muscle insertion in horizontal strabismus remains unclear, and the relationship between horizontal rectus muscle insertion sites and AACE in particular has hardly ever been reported.

The present study investigated whether the horizontal rectus muscle insertion parameters of children with IE would differ according to different ages and compared the horizontal rectus muscle insertion parameters in age-matched patients with IE and AACE.

### 1.1 Patients and methods

This comparative study retrospectively investigated the medical records of 427 consecutive patients, including 166 of IE and 261 of AACE, who underwent horizontal rectus muscle surgery at Zhongshan Ophthalmic Center, Sun Yat-sen University, from June 2014 to August 2020. This study was approved by the Institutional Review Board of the Zhongshan Ophthalmic Center, Sun Yat-sen University, and followed the tenets of the Declaration of Helsinki.

The age-matched patients who met the criteria of IE and AACE were included. Patients were excluded if they had any previous strabismus surgery (except for the current surgery being investigated in the study), fibrosis or palsy of extraocular muscles, systemic or neurological disorders, or a history of ocular trauma. The esodeviation was measured by prism alternative cover test (PACT) at near and distance in the primary position. Strabismus surgeries performed under general anesthesia in this study included unilateral or bilateral MR recession with or without unilateral LR resection. The LID and width of horizontal rectus muscle insertions were measured with surgical caliper intraoperatively. The LID was defined as the distance from the posterior corneal limbus (the transition from the grey cornea to white sclera) to the midpoint of the horizontal rectus muscle insertion. After disinsertion of the rectus muscle, the operated eye was abducted or adducted by fixation forceps at the superior and inferior poles of the insertion site for MR or LR, respectively. Then, the surgeon measured the LID and width, and the other two surgical assistants repeated and confirmed the measurements.

One hundred and fifteen patients with IE under 12 years of age were analyzed separately. A comparative analysis was conducted for patients over 12 years of age, between 51 patients with IE and 261 with ACCE. The IE patients under 12 years of age were analyzed for the correlation between the horizontal rectus muscle insertion parameters and clinical features, including age, refraction, and angle of esodeviation. In patients over 12 years of age, the horizontal rectus muscle insertion parameters and clinical features were compared between IE and AACE, and a correlation analysis was obtained between these factors.

### 1.2 Statistical analysis

Statistical analysis was carried out using the Statistical Package for Social Sciences program (version 22.0, SPSS Inc, Chicago, Illinois, USA). Means and standard deviations, along with ranges, were used to describe the clinical features in Table 1, Table 2. Levene's test, Kruskal-Wallis test, and Mann–Whitney *U* test were performed to evaluate differences between groups. Pearson's correlation coefficient was utilized to detect the correlation of the insertion parameters with age, SE, and the angle of esodeviation.

**Table 1:** Clinical features of the patients with IE (≤12 years old) (n = 115).

Clinical features	Numbers of patients^a^ (male/female)	Mean ± SD (range)
Age at surgery (years)	115 (46/69)	3.83 ± 2.49 (0–12)
MR LID (mm)	111 (45/66)	4.86 ± 0.52 (3.5–6.5)
MR width (mm)	111 (45/66)	7.64 ± 0.96 (6.0–12.5)
LR LID (mm)	29 (13/16)	5.98 ± 0.97 (4.0–7.5)
LR width (mm)	29 (13/16)	7.25 ± 0.87 (5.5–9.0)
SE OD (D)	95 (39/56)	+2.29 ± 1.37 (−1.875 ∼ +6.375)
SE OS (D)	95 (39/56)	+1.92 ± 2.52 (−16.75 ∼ +7.00)
Esodeviation at near (PD)	79 (31/48)	45.61 ± 18.51 (10–100)
Esodeviation at distance (PD)	54 (21/33)	44.01 ± 17.84 (15–86)

IE = infantile esotropia; MR = medial rectus; LR = lateral rectus; LID = limbus-insertion distance; D = diopter; SE = spherical equivalent; OD = right eye; OS = left eye; PD = prism diopter.

a Number of patients who have been measured for the horizontal rectus muscle insertion parameters.

**Table 2:** Comparison of clinical features between the patients with IE and AACE (>12 years old).

Clinical features	Numbers of patients^a^ (male/female)		Mean ± SD (range)		P value
	IE (n = 51)	AACE (n = 261)	IE	AACE	
Age at surgery (years)	51 (26/25)	261 (170/91)	23.49 ± 5.97 (15–45)	25.98 ± 9.58 (13–82)	0.130
MR LID (mm)	40 (17/23)	180 (123/57)	4.98 ± 0.71 (4.0–7.5)	5.00 ± 0.67 (3.0–7.5)	0.851
MR width (mm)	40 (17/23)	180 (123/57)	8.65 ± 1.65 (6.0–12.5)	8.27 ± 1.21 (5.0–12.0)	0.413
LR LID (mm)	36 (17/19)	225 (151/74)	6.57 ± 0.73 (5.0–9.0)	6.28 ± 0.81 (3.5–8.0)	0.099
LR width (mm)	36 (17/19)	225 (151/74)	7.90 ± 1.37 (6.0–10.5)	7.62 ± 1.21 (5.0–11.5)	0.268
SE OD (D)	41 (21/20)	260 (169/91)	−0.44 ± 0.36 (−8.75 ∼ +4.00)	−3.68 ± 2.82 (−12 ∼ +4.25)	0.000∗∗
SE OS (D)	41 (21/20)	260 (169/91)	−0.76 ± 2.73 (−8.75 ∼ +4.25)	−3.34 ± 2.85 (−11.13 ∼ +5.25)	0.000∗∗
Esodeviation at near (PD)	48 (22/26)	258 (169/89)	62.55 ± 30.83 (15–133)	36.80 ± 15.66 (3–103)	0.000∗∗
Esodeviation at distance (PD)	42 (21/21)	259 (170/89)	53.95 ± 31.79 (14–123)	37.97 ± 14.71 (5–104)	0.005∗∗

IE = infantile esotropia; AACE = acute acquired concomitant esotropia; MR = medial rectus; LR = lateral rectus; LID = limbus-insertion distance; D = diopter; SE = spherical equivalent; OD = right eye; OS = left eye; PD = prism diopter.

a Number of patients measured for the horizontal rectus muscle insertion parameters.

## 2 Results

### 2.1 IE (≤12 years old)

**Fig. 1:**
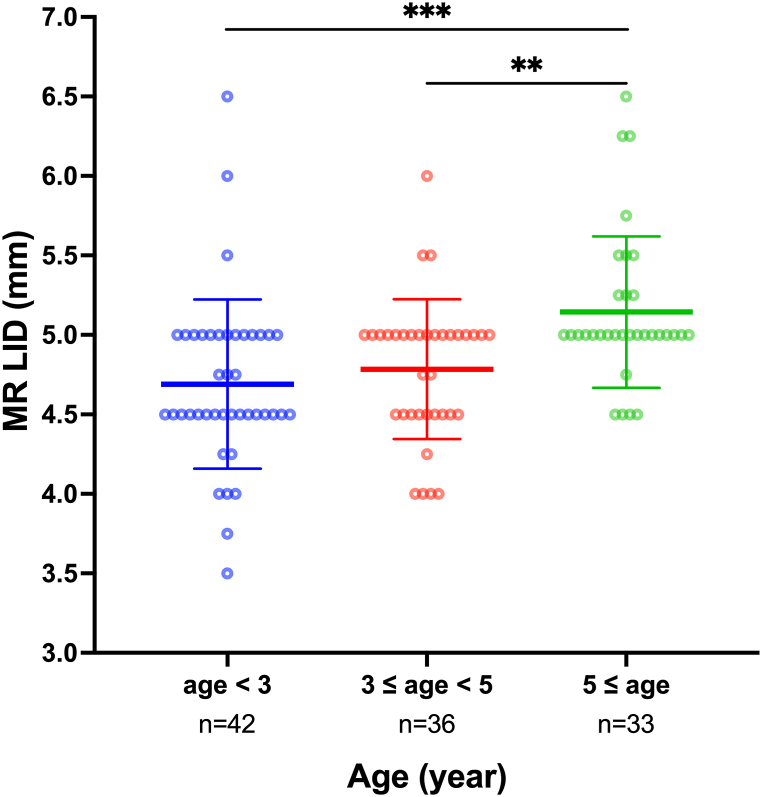
Comparison of the MR LID among different age groups of patients with IE under 12 years of age. The mean MR LID was 4.69 ± 0.53 mm, 4.78 ± 0.44 mm, and 5.14 ± 0.48 mm in the patients with age <3 years old, age of 3–5 years old, and age ≥5 years old, respectively. Statistically significant differences were identified between patients with age <3 years old and age ≥5 years old and between patients with age 3–5 years old and age ≥5 years old.

The clinical features of the patients with IE under 12 years of age are shown in Table 1. The mean MR LID of the patients with age ≥5 years old (5.14 ± 0.48) was significantly larger than the patients with age <3 years old (4.69 ± 0.53) and aged 3–5 years old (4.78 ± 0.44) (Fig. 1).

**Fig. 2:**
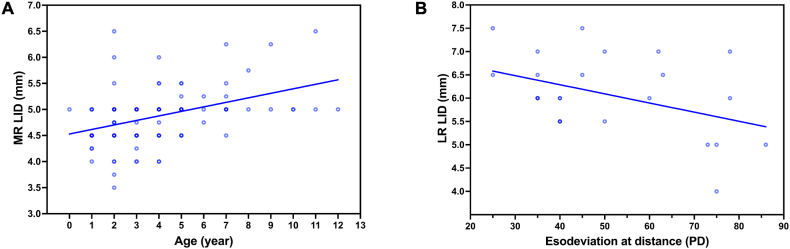
Correlation analysis between insertion parameters and age/esodeviation in patients with IE under 12 years of age. Fig. 2A: Scatter plots showing a positive correlation between age and MR LID (p < 0.0001, r = 0.4164, Y = 0.08666∗X + 4.529). Fig. 2B: Scatter plots showing a negative correlation between the angle of esodeviation at distance and LR LID (p = 0.0365, r = −0.4201, Y = −0.01959∗X + 7.071).

The linear regression showed a positive correlation between age and MR LID (p < 0.0001, r = 0.4164, Y = 0.08666∗X + 4.529, Fig. 2A), and a negative correlation between the angle of esodeviation at distance and LR LID (p = 0.0365, r = −0.4201, Y = −0.01959∗X + 7.071, Fig. 2B).

### 2.2 IE and AACE (>12 years old)

In the age-matched patients over 12 years of age, the clinical features of IE and AACE were compared (Table 2). There was no significant difference between IE and AACE in the mean LID and width of horizontal rectus muscles. The mean SE was more myopic in the patients with AACE than IE, while the mean esodeviation both at near and distance was larger in the patients with IE than AACE.

**Fig. 3:**
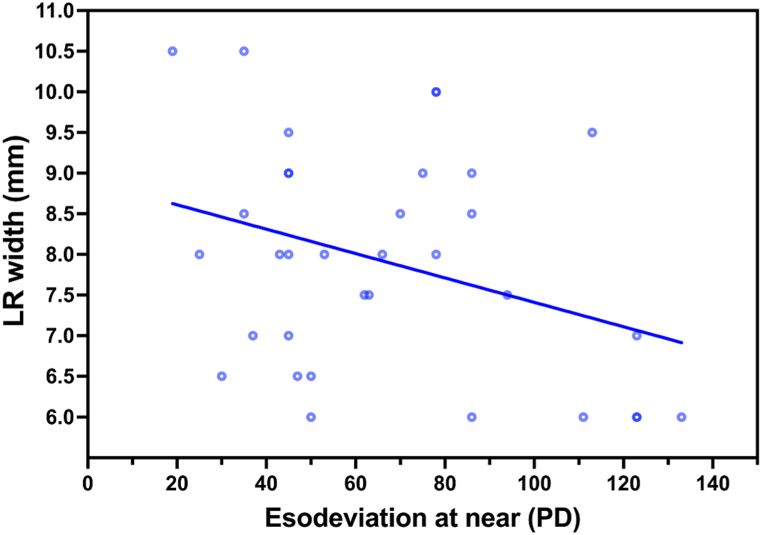
Correlation analysis between the angle of esodeviation at near and LR width in patients with IE over 12 years of age. Scatter plots showing a negative correlation between the angle of esodeviation at near and LR width (p = 0.0496, r = −0.3393, Y = −0.01501∗X + 8.911).

**Fig. 4:**
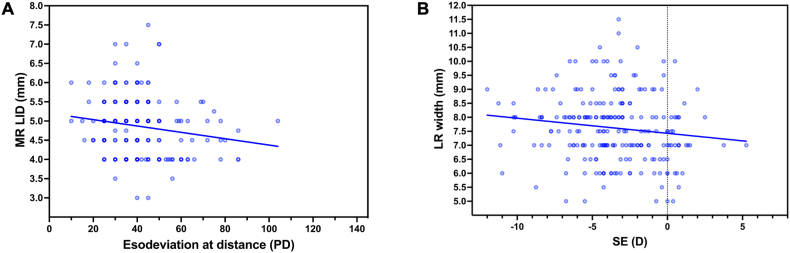
Correlation analysis between insertion parameters and esodeviation/refraction in patients with AACE. Fig. 4A: Scatter plots showing a negative correlation between the angle of esodeviation at distance and MR LID (p = 0.0173, r = −0.1630, Y = −0.008276∗X + 5.202). Fig. 4B: Scatter plots showing a negative correlation between the SE and LR width (p = 0.0443, r = −0.1339, Y = −0.05393∗X + 7.428).

The linear regression showed negative correlation between the angle of esodeviation at near and LR width in the patients with IE (p = 0.0496, r = −0.3393, Y = −0.01501∗X + 8.911, Fig. 3), negative correlation between the angle of esodeviation at distance and MR LID in the patients with AACE (p = 0.0173, r = −0.1630, Y = −0.008276∗X + 5.202, Fig. 4A), and negative correlation between the SE and LR width in the patients with AACE (p = 0.0443, r = −0.1339, Y = −0.05393∗X + 7.428, Fig. 4B).

## 3 Discussion

Extraocular muscles, like other ocular components, undergo major anatomical changes during infancy. In our IE patients with a mean age of 3.83 ± 2.49 years old, the mean MR LID was 4.86 ± 0.52 mm, which is larger than 4.1–4.5 mm in IE patients with an age of about one year old [12,13]. The difference in the MR LID might be induced by age, which was widely recognized that the LID and width of the rectus muscles increase along with age in early childhood [14]. Besides the LID, the widths of rectus muscle insertions in neonates were also roughly 2.5–3 mm narrower than those in adults, and the rectus muscles were adult-like in LID and width by the age of 20 months. Previous studies reported larger LID and width of the MR with increasing age in patients with esotropia [9]. In our IE patients, we identified the positive correlation of the MR LID with age (Fig. 2A), but the LID of LR or the width of MR and LR did not correlate with age. In addition, the MR LID of the IE patients aged≥ 5 years old was larger than in younger patients (Fig. 1), indicating the continuous increase of the MR LID in late childhood. It was reported that the mean LID of the MR is 4.7–4.8 mm in patients with AACE [15,16], which is similar to 5.00 ± 0.67 mm in our AACE patients with about the same age and angle of esodeviation. The LID of MR and LR were reported to be similar in patients with esotropia and exotropia [9]. No significant difference was detected between IE and AACE in the LID and width of MR and LR as well in this study, even though the angle of esodeviation varied greatly between our patients with IE and AACE. There are no reports on horizontal rectus muscle insertions in the patients with IE and AACE has been reported other than the MR LID. The insertion parameters of horizontal rectus muscles detected in this study were shown in Supplementary Table 1 along with the previously reported data.

It is still controversial if the horizontal muscle insertion parameters are different in strabismus compared to the normal population. In a previous study, the LIDs of horizontal rectus muscle insertions were not significantly different between strabismus subjects and controls [10]. However, in other studies, the MR LID was shorter in the patients with AACE than in exotropia [15], and the MR width increased with a greater angle of esodeviation in patients with esotropia [9]. We conducted a correlation analysis between horizontal rectus muscle insertion parameters and several clinical features in different ages and types of esotropia (Table 3). Taking the effect of age on ocular development during childhood into account, we analyzed the IE patients under 12 years of age separately. The results showed that the factors correlated with the angle of esodeviation were different in different age groups of IE patients. In younger IE patients under 12 years of age, the larger angle of esodeviation at distance was correlated with the shorter LR LID; and in older IE patients over 12 years of age, the larger angle of esodeviation at near was correlated with the narrower LR width. The ocular development process probably affected the correlation analysis in the younger IE patients, thus resulting in a paradoxical correlation of a larger angle of esodeviation with shorter LR LID. On the other hand, the correlation of a larger angle of esodeviation with narrower LR width can be explained by a relatively weaker LR force [17].

**Table 3:** Correlation between horizontal rectus muscle insertion parameters and clinical features in patients with IE and AACE.

	IE (≤12 years old)				IE (>12 years old)			AACE (>12 years old)		
	Age	SE	Esodeviation at near	Esodeviation at distance	SE	Esodeviation at near	Esodeviation at distance	SE	Esodeviation at near	Esodeviation at distance
**MR LID**	P	U	U	U	U	U	U	U	U	N
**MR width**	U	U	U	U	U	U	U	U	U	U
**LR LID**	U	U	U	N	U	U	U	U	U	U
**LR width**	U	U	U	U	U	N	U	N	U	U

IE = infantile esotropia; AACE = acute acquired concomitant esotropia; SE = spherical equivalent; MR = medial rectus; LR = lateral rectus; LID = limbus-insertion distance; P = positively correlated; N = negatively correlated; U = uncorrelated.

In AACE patients, we detected that shorter MR LID was correlated with larger esodeviation at distance, and narrower LR width was correlated with higher SE. Recent studies showed that augmented-dosed horizontal muscle surgery was recommended for AACE to avoid surgical under-correction [18,19]. The frequent occurrence of surgical under-correction after classic MR recession and the satisfactory surgical outcome of augmented-dosed MR recession can probably be explained by the shorter MR LID in AACE patients with larger esodeviation. The LR width was suggested to be a useful indicator to estimate the effect of LR recession in patients with intermittent exotropia [17,20]. In this study, the narrower LR width in AACE patients with more hyperopic eyes was probably due to the relatively small diameter of the globe, but had nothing to do with the pathogenesis of the AACE. Intuitively, the smaller the eyeball (i.e., the shorter its axial length), the more anterior the insertion location of the EOMs would be.

To the best of our knowledge, this is the first study comparing horizontal rectus muscle insertion parameters between IE and AACE. This study has some limitations. First, no normal control subjects were included, which could have given the best understanding of the effect of the MR and LR insertion site changes on the misalignment. Second, the strabismus surgeries were performed by more than one surgeon, which might induce bias in the measurements of insertion parameters. Third, the results of this retrospective study only indicated the possible correlation between horizontal rectus muscle insertion parameters and the angle of esodeviation, which may be affected by anatomical changes. Future prospective studies are recommended to reveal more detailed comparisons of different factors, including types of strabismus, function of accommodation and convergence, ocular motility, and surgical outcomes.

In conclusion, this study found no significant difference in the LID or width of horizontal rectus muscle insertions between age-matched individuals with IE and AACE. An increase in MR LID with age was observed in children with IE, which continued in school-aged patients. In addition, the LR width was narrower in IE patients with larger esodeviation, while the MR LID was shorter in AACE patients with larger esodeviation. However, further study is needed to determine whether the changes in horizontal muscle insertion parameters contribute to the pathogenesis of strabismus.

## Funding

None.

## CRediT authorship contribution statement

**Shuyuan Liao:** Writing – original draft, Methodology, Investigation, Formal analysis, Data curation. **Yun Wen:** Methodology, Investigation. **Qingqing Ye:** Formal analysis, Data curation. **Linxing Chen:** Resources, Formal analysis, Data curation. **Tao Shen:** Writing – review & editing, Writing – original draft, Validation, Supervision, Methodology, Investigation, Data curation, Conceptualization.

## Ethics approval and consent to participate

The Institutional Review Board of the Zhongshan Ophthalmic Center at Sun Yat-sen University authorized this study (NO.2015MEKY055). This study followed the tenets of the Declaration of Helsinki. Written informed consents were obtained from all the patients who participated in the study or their parents.

## Data availability statement

The datasets used and analyzed during the current study are available from the corresponding author upon reasonable request.

## Declaration of competing interest

The authors declare that they have no known competing financial interests or personal relationships that could have appeared to influence the work reported in this paper.
