# Supplementary material for: Horizontal rectus muscle insertion sites in patients with infantile esotropia and acute acquired concomitant esotropia
Source: Heliyon. 2025 Jan 16;11(2):e41882. doi: 10.1016/j.heliyon.2025.e41882 (PMC11786817; doi:10.1016/j.heliyon.2025.e41882)
Supplement: Multimedia component 1 [file mmc1.docx]

**Supplementary Table 1: LID and width of horizontal rectus muscles insertions in previous reports.**

| **Reports** | **Population (No.)** | **Age (year)** | **MR LID (mm) *** | **MR width (mm) *** | **LR LID (mm) *** | **LR width (mm) *** | **Deviation (PD)** | **References** |
| --- | --- | --- | --- | --- | --- | --- | --- | --- |
| Apt L.  (1980) (21) | Autopsy (100) | 60.3±16 (21-90) | 5.3±0.7 (3.6-7.0) | 11.3±0.8 (9.6-13.3) | 6.9±0.7 (5.4-8.5) | 10.1±0.8 (8.3-12.5) | - | 21 |
| Barsoum-Homsy M.  (1981) (12) | IE (26) | ≤1 | 4.5±0.57 (3.0-5.5) | NA | NA | NA | NA | 12 |
| Keech RV, et al.  (1990) (13) | IE (20) | 1.3 (0.6-2.6) | 4.1 | NA | NA | NA | ET=55 (35-80) | 13 |
| Kim SH, et al.  (2006) (17) | IXT (37) | 8.9 (7-11) | NA | NA | NA | 8.3±0.72 (6.5-9.5) | XT=19.9±3.17 (16-25) | 17 |
| Kim SH, et al.  (2009) (20) | IXT (26) | 6.3±2.3 | NA | NA | NA | 8.2±0.6 | XT=24.5±1.4 (20–25) | 20 |
|  | IXT (19) | 7.6±4.2 | NA | NA | NA | 8.1±0.6 | XT=33.4±3.5 (30–40) |  |
| Liu L, et al.  (2011) (22) | XT/ET (11/5) | 28.65±8.89 (19-46) | 5.32±0.44 (4.5– 6.1) | NA | 6.58±0.53 (5.5–7.5) | NA | NA | 22 |
| Yun CM, et al.  (2011) (23) | IXT (16) | ＜2 | NA | NA | NA | 7.75±0.34 | XT=31.56±3.97 | 23 |
|  | IXT (20) | 2-4 | NA | NA | NA | 7.68±0.42 | XT=28.50±4.61 |  |
|  | IXT (75) | 5-12 | NA | NA | NA | 8.01±0.39 | XT=28.69±5.14 |  |
|  | IXT (22) | ≥13 | NA | NA | NA | 8.06±0.45 | XT=30.14±4.52 |  |
| Lai YH, et al.  (2012) (10) | Strabismus (123) | 18.5±15.4 (1.5-62.1) | 5.3±0.5 | NA | 6.5±0.7 | NA | ET=36.6±17.4  XT=35.4±16.7 | 10 |
|  | Control (60) | 48.5±15.5 (13.3-81.8) | 5.2±0.9 | NA | 6.3±0.9 | NA | - |  |
| Lee JY, et al.  (2016) (24) | IXT (60) | 9±2 (4-15) | NA | NA | 5.8±0.7 (4.0-7.0) | NA | XT=24.7±5.6 | 24 |
| Niyaz L, et al.  (2017) (9) | ET (75) | 7 (1-55) | 5.7±0.7 | 9.5±0.8 | 6.9±0.8 | 9.8±0.9 | ET=40.2±13.6 (20-75) | 9 |
|  | XT (40) | 18 (2.5-66) | 5.9±0.9 | 9.8±1.1 | 7.1±0.9 | 9.2±0.9 | XT=35.7±11.4 (20-65) |  |
|  | Control (14) | 55 (45-78) | 5.6±1.0 | 9.9±1.3 | 6.7±0.8 | 10.2±1.3 | - |  |
| Cai C, et al.  (2019) (14) | AACE (43) | 21.6±6.6 | 4.8±0.4 (4.0-5.0) | NA | NA | NA | ET=40.5±19.5 (15–90) | 14 |
|  | XT (50) | 21.3±8.6 | 5.4±0.4 (4.5-6.0) | NA | NA | NA | NA |  |
| Honglertnapakul W, et al.  (2020) (25) | Strabismus (110) | 32.2±27.8 (1-90) | 5.0±1.1 (3.0-8.5) | NA | 6.3±0.8 (4.5-8.0) | NA | NA | 25 |
| Jayaraj S, et al.  (2020) (26) | Strabismus (46) | 17.02±7.8 (5-35) | (4.5-6.0) | NA | (5.5-8.0) | NA | NA | 26 |
| Mezad-Koursh D, et al.  (2020) (27) | Strabismus (22) | 34.7±15.5 (18-78) | 6.16±2.0 (4-11.5) | NA | 7.67±2.42 (5.5-14.0) | NA | NA | 27 |
| de-Pablo-Gómez-de-Lian ̃o L, et al.  (2021) (28) | XT (14) | 36.3±16.0 (13–60) | 8.7±2.1 (5.5–12.0) | NA | NA | NA | XT=38.7±16.9 (16–65) | 28 |
| This study | IE (115) | 3.83±2.49 (0 ~ 12) | 4.86±0.52 (3.5 ~ 6.5) | 7.64±0.96 (6.0 ~ 12.5) | 5.98±0.97 (4.0 ~ 7.5) | 7.25±0.87 (5.5 ~ 9.0) | ET=44.01±17.84 (15 ~ 85.71) | - |
|  | IE (51) | 23.49±5.97 (15 ~ 45) | 4.98±0.71 (4.0 ~ 7.5) | 8.65±1.65 (6.0 ~ 12.5) | 6.57±0.73 (5.0 ~ 9.0) | 7.90±1.37 (6.0 ~ 10.5) | ET=53.95±31.79 (14 ~ 122.58) |  |
|  | AACE (261) | 25.98±9.58 (13 ~ 82) | 5.00±0.67 (3.0 ~ 7.5) | 8.27±1.21 (5.0 ~ 12.0) | 6.28±0.81 (3.5 ~ 8.0) | 7.62±1.21 (5.0 ~ 11.5) | ET=37.97±14.71 (5 ~ 103.66) |  |

* LID and width of horizontal rectus muscles were measured with surgical caliper during strabismus surgery.

LID = limbus-insertion distance; PD = prism diopter; IE = infantile esotropia; IXT = intermittent exotropia; AACE = acute acquired concomitant esotropia; ET = esotropia; XT = exotropia; NA = not available.
